# Investigation on Some Algal Extracts as Appropriate Stabilizers for Radiation-Processed Polymers

**DOI:** 10.3390/polym14224971

**Published:** 2022-11-16

**Authors:** Traian Zaharescu, Carmen Mateescu

**Affiliations:** INCDIE ICPE CA, 313 Splaiul Unirii, 030138 Bucharest, Romania

**Keywords:** SIS, stabilization, *Chlorella vulgaris*, *Spirulina platensis*, *Ascophyllum nodosum*, chemiluminescence

## Abstract

This study presents the appropriate solution, algal extracts, for the improvement of polymer durability when the material is subjected to acute oxidation damage. The investigated support, styrene–isoprene–styrene (SIS), is modified by three algal extracts: *Chlorella vulgaris*, *Spirulina platensis*, and *Ascophyllum nodosum* (Kelp) with a low concentration (1 wt%). The presence of polyhedral oligomeric silsesquioxane (POSS) ensures the growth of stability with respect to the pristine polymer. The thermal performances of the host polymer, indicated by chemiluminescence, reveal the essential contribution of an additive to the improvement in oxidation strength. The stability of the polymer adjusted by algal extracts is proved by the activation energy values, which increase from 49 kJ mol^−1^ to 89 kJ mol^−1^ for the same polymer modified with *Ascophyllum nodosum*. This main important characteristic is the consequence of the highly efficient activity of the polyphenol components of algal extracts and the effect of the three natural additives on the favorably changed kinetic parameters (oxidation induction time and onset oxidation temperature). The exposure of the polymer matrix to the damaging action of γ-rays does not affect the proper contributions to the fast delay in material ageing. The irradiation of 100 kGy, a usual technological dose, may be successfully applied in the radiation processing of a polymer stabilized with algal extracts due to the efficient protection of the additive as the chain-breaking agents.

## 1. Introduction

The stabilization of polymer materials is a basic condition for their long-term durability. The best explanation of the initiation of degradation by the stressors with extended acting periods and intensities involves not only structural limitations, but also the presence of efficient protectors [1,2]. At present, there are several options for the improvement of polymer material strength: crosslinking by reactive processing [3] or high-energy irradiation [4], addition of natural extracts [5] and fillers [6], or both of them [7]. These alternatives present specific mechanisms by which the oxidation chains are broken [8]. The selection of the solution for the structural safeguard depends on the potential availability of an additive for the inactivation of intermediates in the different stages of the propagation chain. The consumption and the migration of protectors are some principal limitations by which organic structures (hindered phenols and amines) differ from inorganic fillers (oxides, clays, and polyhedral silicates). However, their coupling may offer a real key for the manufacture of high-performance polymeric materials.

The stability of polymers is a major concern that requires an efficient action starting from the early stages of degradation [9]. The materials permanently subjected to intense stressors—such as multiple cycles of heating/cooling, sun, heat, light, and high-energy radiation—must possess a certain inhibition factor that delays the progress in the oxidation state. The modification of the formulation by the addition of a suitable compound has to scavenge free radicals in order to eliminate them from the degradation process. The ubiquitous oxidation may be avoided by the association of a stability protector with the controlled molecular scission [10]. The sources of active principles for the improvement in plastics’ durability may be easily found and obtained from various types of algae [11,12,13,14,15]. Unfortunately, the concentration of antioxidant components in the algal extracts depends strongly on environmental conditions, the harvesting season, and growing factors [16,17]. The great interest in the optimal concentration of antioxidant compounds in algae also concerns detailed investigations on appropriate extraction procedures, which are pertinently correlated with technological stability and separation efficiencies.

The extension of applications for algal extracts is related to the high content of antioxidants, which offer healthy handling and implications [18]. Several ranges of applications are concerned with the processing of algal extracts: biotechnologies [19,20], production of biomethane [21], and food preparation and preservation [20]. The great potential of microalgal extracts is easily confirmed by the deep interest in the production of antioxidant products [22], which are largely used in food industry, medicine and domestic activities, such as painting and healthy cleaning.

The stabilization effects promoted by algal extracts are related to the presence of active components that recommend them as suitable additives for the hindering of oxidation in organic compounds like polymers. *Chlorella vulgaris* and *Spirulina platensis* microalgae were grown organically in a protected environment in an open pond owned by a producer in Spain and were purchased in the form of powder from the pharmacy. The chemical composition of the *Chlorella* sp. (*w*/*w*%) is fats 11%, of which 2% are saturated forms; carbohydrates 25%, of which sugars are 1%; proteins 56%; and natrium chloride 0.1%. The powder of Spirulina sp. microalgae contains fats 8%, of which 1% are saturated structures; carbohydrates 13%, of which sugars are 2%; proteins 67%; and natrium chloride 1.1%. *Ascophyllum nodosum* macroalgae was grown in an ecological culture in the coastal waters of the northeastern Atlantic Ocean. After collecting, drying, and grinding the macroalgae, the powder was distributed for sale as a food supplement. The chemical composition of the macroalgae used for this experiment (*w*/*w*%) is fats 5%, of which 2% are saturated forms; carbohydrates 71%, of which sugars are 4%; proteins 7%; and natrium chloride 0.63%. The stabilization mechanism is assimilated with the action of polyphenols. The chain breaking is the contribution of algal extracts that diminish the oxidation rate in the propagation stage of oxidative degradation [16,23]. 

The applications of microalgal extracts in the improvement in polymer strength against oxidation have been scarcely studied [24,25,26,27]. The abundance of antioxidants in algal mass [28] recommends the algal masses as the veritable source of oxidation inhibitors [29]. Even though the period of harvesting raises the problem of the concentration of active components [28,30,31], the quality of oxidation protection must not be disregarded [32].

The investigation of the antioxidant activities of algal extract has been scarcely reported. In spite of their high efficiency [33], algal extracts as well as fresh plants are usually considered appropriate food supplements [34]. An interesting survey on the applications of algal extracts was already published [35], where the benefits of their antioxidant content are clearly revealed. However, the most popular purpose of algal extracts remains medical and health care applications [36], by which mankind is able to maintain an appropriate level of good condition. However, the natural antioxidants existing in algal biomasses present a safety guarantee when they are added to various materials that recommend them as the main factor involved in the life of high-performance polymers. They expand technological methods of manufacturing ecological products. The substitution of classical antioxidants (hindered phenol and amine compounds) with high-efficiency natural extracts is a great opportunity for the preservation of human health and for the limitation of hazards during the production of industrial products. Furthermore, natural extracts are a great opportunity to achieve the increased stability required by recycled polymers, especially in radiation treatment. The production price is another characteristic that opens the market, offers a pertinent incentive for manufacturers and indicates a suitable way for producing good quality as a permanent customer demand.

Interest in the applications of algal extracts is appropriately stimulated by the contribution of active components to the scavenging of free radicals from their oxidation. Various structures with antioxidant features—such as polyphenols and tocopherols and especially vitamin E, pigments, carotenoids, chlorophylls, and phycobilins [37]—act successfully on the breaking down of the degradation chain initiated by peroxyl radical [38].

This study aims to bring to light a less-exploited application of algal biomass in the advanced polymers industry and to investigate the antioxidant capacity of the *Chlorella vulgaris* and *Spirulina platensis* microalgae and *Ascophyllum nodosum* macroalgae on the styrene-isoprene-styrene triblock copolymer in association with an inorganic filler, polyhedral oligomeric silsesquioxane, to create an oxidation-resistant polymeric matrix and to confer improved thermal and oxidation stability.

## 2. Materials and Methods

The algal extracts (*Chlorella vulgaris*, *Spirulina platensis* and *Ascophyllum nodosum*/Kelp) were purchased from the pharmacy. They were used as received products. polyhedral oligomeric silsesquioxane (POSS) was produced by Sigma Aldrich (USA) as powder, whose average-size particles were 200 nm. Styrene–isoprene–styrene (SIS) was produced and shipped by KRATON, USA.

The samples were prepared by the addition of a similar amount (0.5 wt%) of each additive to a chloroform solution of SIS. For chemiluminescence (CL) measurements, the small aliquots of each modified solution were poured into aluminum pans. Then, the wet samples were evaporated at room temperature and dry, thin layers were obtained.

The γ-exposure was achieved in air at room temperature in an irradiation system, Ob Servo Sanguis (Hungary), by applying the continuous rotation of the hosting can for homogenous exposure. Dose rate was 0.5 kGy h^−1^. The samples were analyzed soon after the end of irradiation periods, avoiding any chemical modification.

**Scheme 1:**
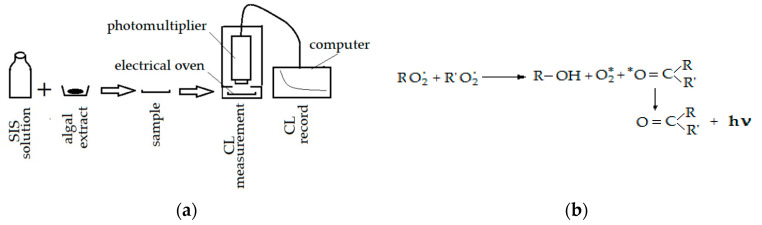
(**a**) CL measurement chain and (**b**) CL emission for degradation survey.

The chemiluminescence (CL) determinations were performed with a specialized device, LUMIPOL 3, produced by the Institute of Polymers, Slovak Academy of Science, Bratislava. The method based on the following process was described in detail in a previous report [39]. Scheme 1 presenting the experimental background of CL measurements is placed below. The proportionality between the concentration of peroxyl radicals, the chain careers in the oxidation mechanism, and the number of emitted photons allows for the correct evaluation of the progress in polymer degradation or stabilization. The formation of peroxyl radicals by the reaction of free molecular fragments with diffused oxygen is the source for the next degradation stage, in which one of the reactions is their recombination, followed by the emission of a CL photon for each individual process. While the increase in temperature is adjusted in nonisothermal measurements by a controller unit according to a previously established heating rate, the isothermal determinations are accomplished by means of a thermosetting switcher whose temperature value is carefully stated in relation to the degradation rate and the type of polymer. The normalization to the mass unit of Cl intensity values is required for the reliable comparison of results.

Both measurement procedures, nonisothermal and isothermal, were applied for the characterization of the modification that occurred in the oxidation states of the samples, either by varying temperature or by elapsing degradation time, respectively. While the heating rate of 10 °C min^−1^ was applied for nonisothermal measurements, the isothermal determinations were conducted at pertinent temperature range (120–150 °C), where the records could be accomplished with convenient recording rates. The measurements were taken immediately after the end of irradiations. 

## 3. Results

### 3.1. Isothermal Chemiluminescence

**Figure 1:**
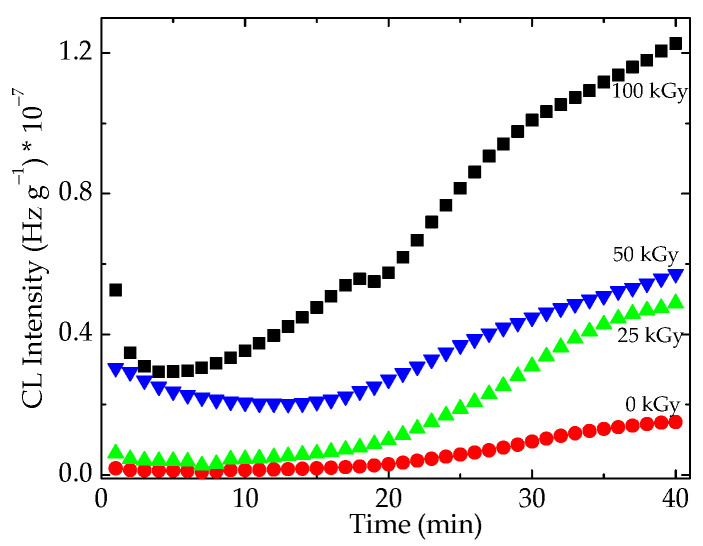
Isothermal chemiluminescence spectra obtained on the pristine SIS samples subjected to different exposure doses. Testing temperature: 130 °C.

The advanced degradation of SIS was obtained by the exposure to an energetic action of γ-rays that represents an accelerated ageing. The early evolution of oxidation that occurred in polymer material is illustrated in Figure 1. If the damage to the SIS structure takes place smoothly in nonirradiated samples, the γ-irradiated patterns show an increase in the photon emission in the shortened periods when the exposure doses are extended. This behavior indicates an indispensable requirement: the presence of a suitable compound that efficiently delays this decay [40].

**Figure 2:**
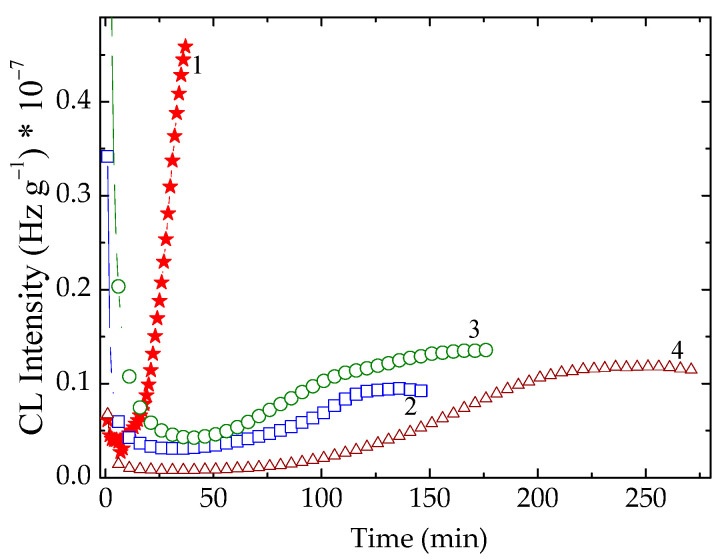
Isothermal chemiluminescence spectra obtained on the unirradiated SIS samples modified with different algal extracts. Testing temperature: 120 °C. (1) Free of stabilizer, (2) *Chlorella vulgaris*, (3) *Spirulina platensis*, (4) *Ascophyllum nodosum*.

As it was seldom demonstrated, the synthesis compounds, such as hindered phenols, are able to protect polymers against the inevitable oxidation [41]. This improvement in material durability seems to indicate that the substitution of this kind of antioxidant with other natural compounds that behave similarly is a smart implementation [42]. The use of algal extracts provides a good chance for the long-term stabilization of polymers (Figure 2).

As it may be noticed from Figure 2, the efficiency of these additives is impressive and the material that results from these compositions presents an extended stability. While the prostine SIS is totally degraded, these modified formulations are at the start of their oxidation. The content of active components dictates the activity of additives and the results appear accordingly.

**Figure 3:**
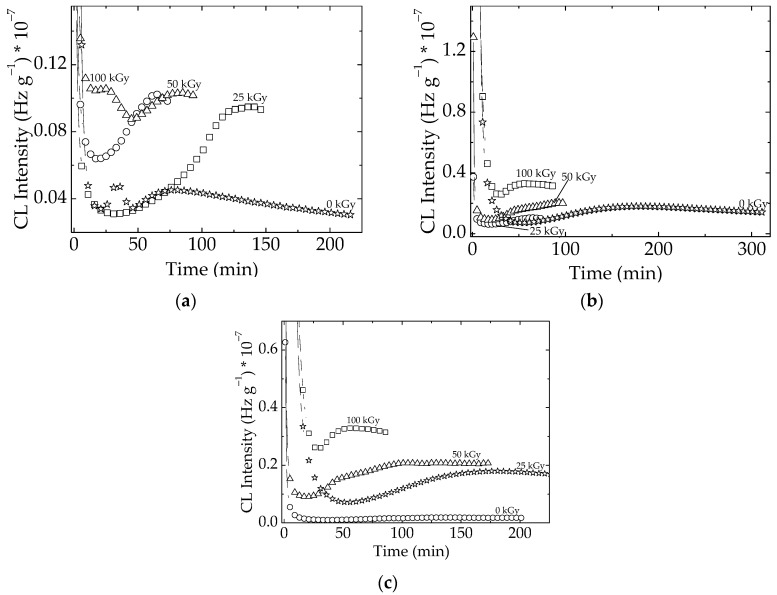
Isothermal chemiluminescence spectra of irradiated SIS samples. Testing temperature: 110 °C. (**a**) *Chlorella vulgaris*, (**b**) *Spirulina platensis*, (**c**) *Ascophyllum nodosum*.

The radiation resistance of these antioxidant extracts is demonstrated in Figure 3. It would be expected that the material stability would drop sharply because the increase in dose from 25 kGy to 100 kGy would affect the structure of the polymer constitution.

Similar CL curve shapes were reported for other stabilized systems, whose stabilization mechamism is based on the breaking degradation chain [43], but the differences highlight the structural efficiency due to the polyphenolic compositions [44]. This characteristic is mentioned in detail for seaweeds destined for the preparation of efficient drugs [45].

**Figure 4:**
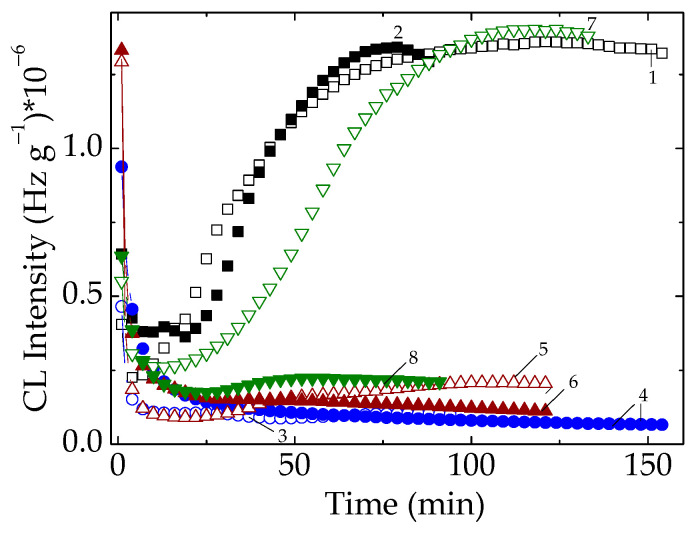
Isothermal CL spectra recorded at 120 °C on the SIS samples modified by the presence of various additives. Irradiation dose: 100 kGy. (1) Neat polymer; (2) SIS/POSS; (3) SIS/*Chlorella vulgaris*; (4) SIS/*Chlorella vulgaris*/POSS; (5) SIS/*Spirulina platensis*; (6) SIS/*Spirulina platensis*/POSS; (7) SIS/*Ascophyllum nodosum*; (8) SIS/*Ascophyllum nodosum*/POSS.

In spite of the strong destructive action of *γ*-radiation, the presence of protective additives like algal extracts and POSS powder supports the peculiar effects on the delay of oxidative degradation. In Figure 4, the contributions of various algal extracts in combination with POSS can be learned. The very low CL intensities resulting from the initiation of stabilization are relevant even at 100 kGy, a technological dose, when the degradation of the polymer support reaches a notable degree. The excellent responses of the most-investigated formulations may characterize them as protection solutions with special potential.

### 3.2. Nonisothermal Chemiluminescence

**Figure 5:**
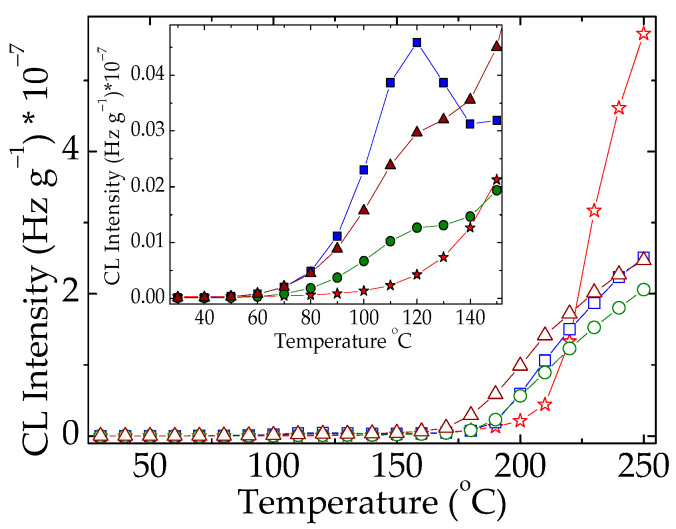
Nonisothermal CL spectra recorded on neat SIS irradiated at various doses. Heating rate: 5 °C min^−1^. Stars: 0 kGy, circles: 25 kGy; triangles: 50 kGy, squares: 100 kGy.

The degradation accelerated by *γ*-irradiation may indicate the material behavior under severe conditions of operation. The deailed analysis of material performances subjected to early ageing (Figure 5) shows the intimate transformation, which cannot be observed through a rough inspection.

**Figure 6:**
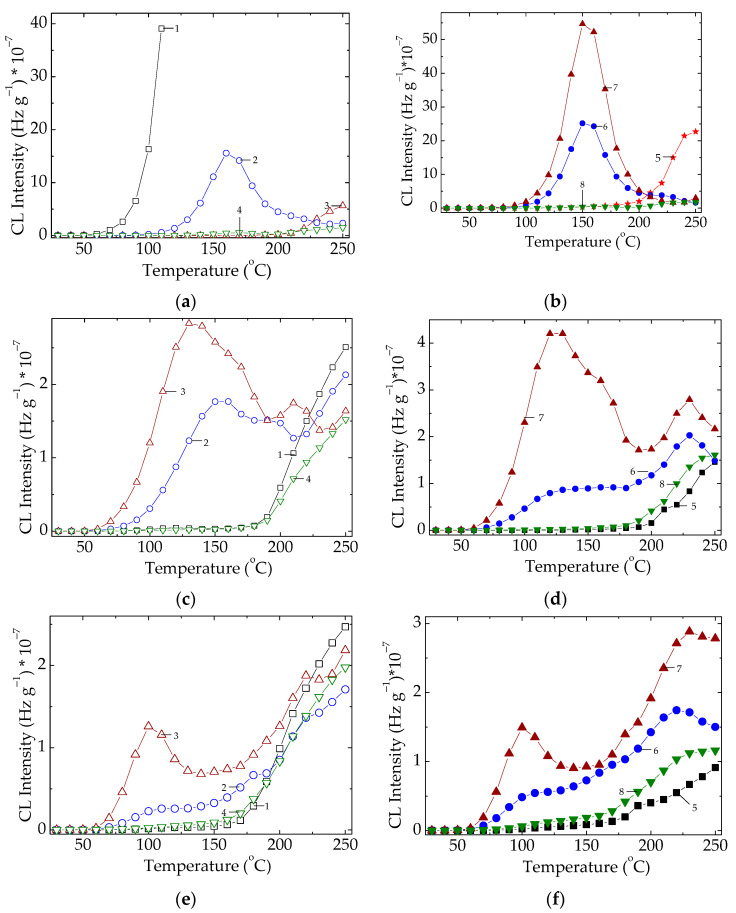
Nonisothermal CL spectra for modified SIS patterns dubjected to different *γ*-irradiation doses. (**a**,**b**) 0 kGy; (**c**,**d**) 25 kGy; (**e**,**f**) 100 kGy. Heating rate: 10 °C min^−^^1.^ (1) neat polymer; (2) SIS/*Chlorella vulgaris*; (3) SIS/*Spirulina platensis*; (4) SIS/*Ascophyllum nodosum*. (5) SIS/POSS; (6) SIS/*Chlorella vulgaris*/POSS; (7) SIS/*Spirulina platensis*/POSS; (8) SIS/*Ascophyllum nodosum*/POSS.

The evolution of oxidation takes place differently for the samples containing POSS and its couples with algal extracts. This means that a synergistic effect appears and the products containing these couples present an evident increase in durability. The oxidation advances differently as the heating process is developed with various rates (Figure 6). The composition plays an important role, because the polymer is unlikely to be protected and the interaction between SIS macromolecules and additive particles has various degrees. The instability of samples is characterized by the maximum CL intensity, which demonstrates the formation of a hydroperoxide. The high is proportional to the amount of ROOH, which is decomposed later on. At a low irradiation dose (25 kGy, the dose applied when sterilization is achieved), only the sample containing Ascophyllum nodosum is stable up to 185 °C and the absence of an oxidation peak at 155 °C may be noticed. The longer radiation treatment obtained at 100 kGy reveals the formation of hydroperoxides at a somewhat lower temperature (100 °C) due to the fragmantation of polymer molecules and the disponibility of intermediates to an earlier oxidation. If the formation of oxidized intermediates has a maximum at 155 °C in unirradiated materials, where the vulnerable bonds are scissed, the free radicals are more easily converted into degradation products at lower temperatures. The greater the exposure doses, the lower the temperature of oxidation.

The presence of inorganic particles of POSS influences the degradation in the material bulk. For the irradiated specimens containing Ascophyllum nodosum, the degradation is retarded to a larger extent and the contribution of POSS is determinant.

## 4. Discussion

The protection activity of additives against oxidation may be explained by their interaction with free radicals, the molecular segments involved in oxidative degradation [46]. The lifetime of ageing polymer materials is strongly dependent on their structural features and composition. Control of the degradation rate is a basic requirement for the qualification of any compound that shows antioxidant characteristics. Through the valuable classes of oxidation delayers, natural products gain top positions in the range of polymer stabilizers [47]. As demonstrated previously, these types of compounds may be beneficially associated with other inorganic structures in order to increase their efficiency [7].

**Figure 7:**
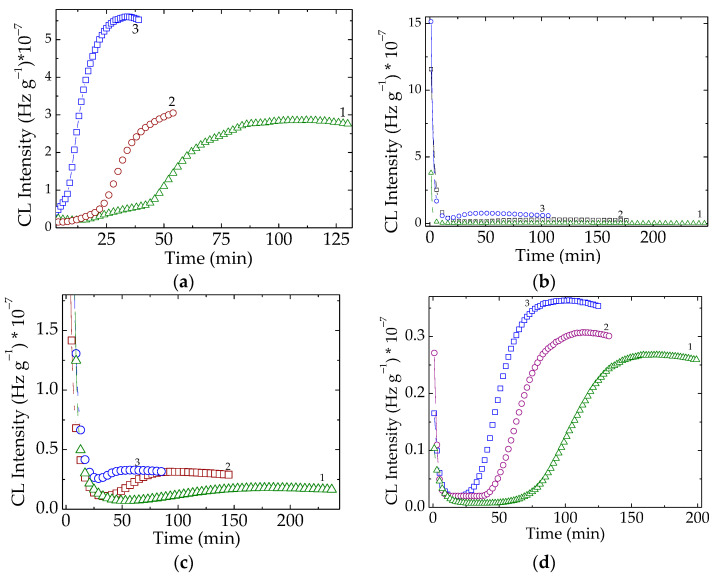
Isothermal CL spectra recorded on unirradiated SIS/algal extract samples. (**a**) Free of additive; (**b**) *Spirulina platensis*; (**c**) *Chlorella vulgaris*; (**d**) *Ascophyllum nodosum.* Testing temperatures: (1) 130 °C; (2) 140 °C; (3) 150 °C.

The oxidation of SIS that starts with the scission of residual double bonds and bonds associated with ternary carbon atoms can be effectively retarded and slowed down by algal extracts, which contain blends of polyphenols and other stabilizing compounds [26]. The increase in the number of free radicals due to energetic ageing (Figure 1) requires the prevention of their reactions with diffused oxygen. The scavenging activity of studied stabilizers is characterized by the extension of oxidation induction times (Figure 2) and onset oxidation temperatures (Figure 6), the main kinetic parameters that demonstrate the capacity of additional compounds to assist stability improvements. The qualification of oxidation degree is appropriately achieved by the chemiluminescence investigation, because the progress of degradation is suitably monitored. The results may be interpreted starting from the capacity to break autocatalytic degradation chains and block the reactivity of intermediates with respect to penetrated molecular oxygen. The three algal extracts present remarkable stabilization activities, which are arranged in the following order (Figure 7):

*Ascophyllum nodosum* > *Chlorella vulgaris* > *Spirulina platensis* > free

The OIT obtained from the isothermal CL determinations allowed for us to calculate the values of activation energies involved in the oxidation process (Table 1). The ascendant figures obtained in the presence of algal extracts demonstrate the beneficial consequences brought about by them in the protection action. The efficiency is related to the antioxidant loadings, which are the unique traits of the original sources [47].

The increase in the stabilization activities is obtained through the co-operation between the two types of compounds: the natural antioxidants from algal powders and the inorganic lattice of POSS. If the two stabilizers act differently, they are completing each other and the overall effect is the increase in material stability.

**Figure 8:**
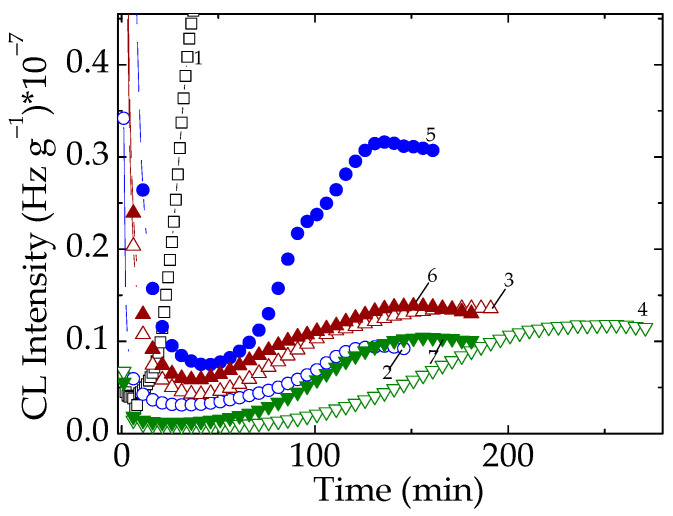
Isothermal CL spectra recorded on the SIS stabilized with various active protectors against oxidation. Irradiation dose: 25 kGy. Testing temperature: 130 °C. (1) Neat polymer; (2) *Chlorella vulgaris*; (3) *Spirulina platensis*; (4) *Ascophyllum nodosum*; (5) SIS/*Chlorella vulgaris*; (6) *Spirulina platensis*/POSS; (7) SIS/*Ascophyllum nodosum*/POSS.

The most important feature that must be revealed is the delayed activities in the irradiated material. It is remarkable that the interaction of the studied additives and the polymer matrix is not influenced by the decay of the radical scavengers, which becomes an essential characteristic of radiation technologies, especially radiation sterilization (Figure 8).

The selection of the SIS formulation to encourage the long-term utilization of items manufactured by radiation processing is sustained by the enhanced stability created when contained macromolecules are split during radiolysis.

## 5. Conclusions

This paper presents the improvement in the thermal and radiation strengths reached by a polymer material, styrene–isoprene–styrene triblock copolymer, in the presence of algal extracts. The association of the latter with an inorganic filler, polyhedral oligomeric silsesquioxane, creates proper couples for the protection of the polymer matrix from oxidation. The applications of these materials are supported not only by the natural source of stabilizers, but also by the substantial extension of oxidation induction times revealed at 130 °C: from 32 min, when oxidation occurs in pristine polymer, to 53 min, 83 min, and 78 min for the degradation of SIS in the presence of *Spirulina platensis*, *Chlorella vulgaris* and *Ascophyllum nodosum*, respectively. This suggests that the stabilization effect endures longer when the manipulation of materials is done in the room-temperature range or a little higher. The co-operation between the studied algal extracts and POSS increases the durability of products due to the coupling activities in the polymer substrate, demonstrated by the lower positions of CL isothermal curves with respect to the evolution of oxidation controlled only by algal extracts. The stability determinations involving nonisothermal CL spectroscopy show the highest protection effect of *Ascophyllum nodosum* in comparison to the other two natural products (*Spirulina platensis* and *Chlorella vulgaris*), which confirms the benefits of its selection for the manufacture of ecological and healthy products. The strongest proof that supports the preference of algal extracts over synthetic antioxidants is the pertinent values of activation energies required for the oxidation of a host polymer. If neat material needs only 49 kJ mol^−1^, this kinetic parameter attains much higher figures that are 1.5–1.8 times larger for the same polymer improved by algal extracts.

The present assay is a starting point for the extensive usage of algal extracts as friendly products, especially in the production of beverage bottles and packaging sheets and boxes. The compatibility of plastics and algal extracts is a pertinent signal for the extension of application ranges. The addition of algal extracts is a pertinent technological option for the applications of radiation processing in the manufacture of plastics products or in the recycling of polymer waste.

The presented results may be taken into consideration for further investigations of the stabilization of various classes of polymers, because algal extract powders enable increased operation periods with appropriate safety procedures, doubled by their beneficial properties with respect to people.

## Figures and Tables

**Table 1:** Activation energies for the thermal oxidation of SIS/algal extracts samples.

Sample	Oxidation Induction Time (min)			Correlation Factor	Activation Energy (kJ mol^−1^)
	130 °C	140 °C	150 °C		
free	32	24	15	0.99682	49
*Spirulina platensis*	53	40	25	0.98756	53
*Chlorella vulgaris*	83	50	28	0.99861	76
*Ascophyllum nodosum*	78	35	20	0.99858	89
